# Cantonese media promotes Chinese cultural identification: structural equation modeling based on Malaysian Chinese

**DOI:** 10.3389/fpsyg.2023.1217340

**Published:** 2023-08-24

**Authors:** Nian Liu, Tongyu Chen, Yuqing Peng, Ying Xie

**Affiliations:** ^1^School of Public Administration, Guangzhou University, Guangzhou, China; ^2^School of Arts (Journalism), Monash University, Melbourne, VIC, Australia; ^3^School of Journalism and Communication, Guangzhou University, Guangzhou, China

**Keywords:** Cantonese media, Chinese cultural identification, ethnic minorities, structural equation model, mediation

## Abstract

**Introduction:**

Language media from one’s hometown is an important means of maintaining cultural identification, especially for minorities. Cantonese media plays an active role in shaping the Chinese cultural identification of ethnic Chinese all over the world. To date, few researchers have undertaken quantitative empirical analyses of the mechanism through which Cantonese media influences cultural identification.

**Methods:**

Using data from 642 Malaysian Chinese, this study established a structural equation model with the partial least squares method.

**Results:**

We found that the emotional affinity of ethnic Chinese to Cantonese media can influence identification with Chinese culture through the perceived value of Cantonese media and cognition of Chinese culture. The perceived value of Cantonese media (*IE* = 0.208) and cognition of Chinese culture (*IE* = 0.068) play partial mediation roles. Meanwhile, emotional affinity to Cantonese media influences cognition of Chinese culture (*IE* = 0.069) through the chain mediation of perceived value of Cantonese media and cognition of Chinese culture. Age has a partial moderating effect in the structural equation model. Compared with minors, adults’ emotional affinity to Cantonese media can eventually influence identification with Chinese culture (*TE_diff_* = 0.126) more strongly through several mediation paths.

**Discussion:**

The study suggests a need to cultivate the emotional affinity of ethnic Chinese to Cantonese media, improve the multidimensional values of Cantonese media, and endow Cantonese media with functions of cultural dialog and knowledge transmission. The international transmission of Cantonese media could play a vital role in building a cultural community for ethnic Chinese globally.

## 1. Introduction

Language is an important medium of national identity. If the language of an ethnic group or diaspora is restricted and literacy is disrupted, the cultural identity of the ethnic group may be lost (Phinney et al., 2001). In contrast, media relying on the native language can preserve minority cultures. In the information age, the rapid dissemination of new media content greatly affects the self-perception of ethnic groups. For example, new media content representing black African culture has enhanced the sense of belonging for the dispersed black African ethnic population (Cottle, 2000). Although dispersed ethnic groups must make great changes in the process of adapting to a new culture, the values, religious beliefs, and traditional customs of many indigenous cultures will not be fundamentally changed by ethnic group migration (Safran, 2004).

Chinese dialects are an emotional link between China and Chinese citizens overseas. Cantonese, a common Chinese language in Guangdong, Hong Kong, Macau, Southeast Asia, and globally, is a tool for Chinese communication and a carrier of cultural memory and nostalgia. Population estimates released by the Department of Statistics Malaysia (2022) indicate that Chinese accounted for 22.8% of Malaysia’s 32.7 million population, or about 7.4556 million people. Outside of China and Singapore, Malaysia has the highest proportion of Chinese people, the highest concentration of overseas Cantonese media, and a strong ethnic Chinese cultural identity. Thus, studies of Cantonese media and Chinese cultural identity are frequently conducted in Malaysia.

Scholars emphasize the role of Cantonese media in shaping cultural identity (Zeng and Agha, 2021). However, the existing research lacks a quantitative empirical analysis of the mechanism through which Cantonese media affects cultural identity. This paper uses structural equation modeling to explore the impact of Cantonese media on Chinese cultural identity and clarify the psychological path of the influence of native-language media on the cultural identity of Malaysian Chinese. The findings have important academic value and practical significance.

## 2. Literature and hypotheses

### 2.1. Cantonese media and Chinese cultural identification

Cultural identification refers to recognition of the common culture between people or between individuals and groups. Overseas Chinese typically retain a strong emotional attachment to their hometowns and cultural identity. This phenomenon is likely to continue while Chinese culture remains diverse, vital, and attractive, and is promoted through the establishment of effective media communication mechanisms (Gungwu, 1993). Imagined community theory emphasizes that the media, particularly print media, influences how citizens construct the imagination of a community (Anderson, 2005). We further derive from this theory that mass media has created imagined communities of diasporas, with language as a mediator.

The classic theory of cultural identity and diaspora indicates that native-language media plays a significant role in constructing group cultural identity in a diaspora (Hall and Ghazoul, 2012). With rising migration worldwide, the question of identity has become increasingly salient in modern society. Struggling with different cultural frames, diasporas’ cultural identities are constructed through the interaction between similarity and difference in the process of daily life media use (Xie, 2005). According to cultural memory theory, “Human memory is ‘embodied’ in living personal memories and ‘embedded’ in social frames and external cultural symbols (e.g., texts, images, and rituals) that can be acknowledged as a memory function insofar as they are related to the self-image or ‘identity’ of a tribal, national, and/or religious community” (Assmann, 2011). The storage and inheritance of memory largely depend on the media in the context of global flows. Native-language media can connect discrete ethnic groups with their original hometowns (Assmann, 2012).

Across large swaths of Chinese overseas communities in Southeast Asia, native speakers view Cantonese as a vital and inseparable part of their cultural identity (Gungwu, 1988). Cantonese media consists of forms, including television, radio, cinema, newspapers, magazines, websites, and other online platforms. Emotional affinity to Cantonese media refers to individuals’ degree of fondness toward this media. Love for Cantonese media is clearly evident among Malaysian Chinese, with Cantonese movies performing well at the box office in Malaysia and Cantonese songs widely sung. Indeed, Cantonese New Year movies and songs have been flourishing for decades (Trémon, 2022). With respect to usage of Cantonese media, perceived value refers to users’ comprehensive evaluation of its overall worth (Wang et al., 2021). Many scholars have explored the perceived value of media from different angles, including entertainment value (Abbas Naqvi et al., 2020), cultural value (Albert, 2021), instrumental value (Swaid and Wigand, 2012) and social value (Wan and Chew, 2013). Cantonese media has been found to have entertainment and cultural value, and plays an important role in shaping the imagined community of cultural China (Chan and Khaild, 2020). Cantonese media has profoundly affected overseas Chinese, linking Chinese cultural memory and cultural identity across time and space, shaping the transnational Chinese imagination, and constructing a transnational Chinese cultural community (Ng and Cavallaro, 2019).

Cantonese media has formed a specific Chinese cultural field for audiences. Through media interpretation and unique scenes, audiences share a strong sense of identity and pride in their culture (Jerome et al., 2022). Cantonese media can enhance the cultural identity of Chinese ethnic groups. Daily use behaviors ensure the cultural identity model of the social structure is embedded in the collective memory of generations of ethnic Chinese in Malaysia, shaping the cultural emotions of ethnic Chinese living there (de Sousa, 2021).

Specific to the construction of cultural identity through the use of Cantonese media in Malaysia, studies have focused on Cantonese media, language and cultural identity, Cantonese media and the construction of Chinese social networks, and Cantonese media and the construction of Chinese ethnic culture (Ong and Ben-Said, 2022). Research on the role of Cantonese media in the construction of cultural identity has mainly focused on Hong Kong popular culture, represented by Hong Kong film and television dramas and pop music. Cantonese martial arts films in Hong Kong are a source of entertainment for Malaysian Chinese and a link between real life and nostalgic imagination (Khoo, 2014). Hong Kong films have influenced the cultural identity of overseas Chinese. The films present the theme of immigration and a diasporic narrative, sharing the globally dispersed cultural memories of Chinese and further shaping the global Chinese cultural community (Chu, 2017). Cantonese pop music has developed rapidly since the early 1970s to become popular in Malaysia. Thus, the perceived value of Cantonese media for overseas Chinese is widely recognized, and has promoted the global communication of Chinese society.

Accordingly, we proposed the first set of hypotheses:

*H1*: Emotional affinity to Cantonese media positively affects Chinese cultural identification. When individuals like Cantonese media, they are more likely to identify strongly with Chinese culture.

*H2*: The perceived value of Cantonese media positively affects Chinese cultural identification. When individuals place a high perceived value on Cantonese media, they are more likely to identify strongly with Chinese culture.

*H3*: Chinese cultural cognition positively affects Chinese cultural identification. When individuals understand Chinese culture, they are more likely to identify strongly with Chinese culture.

### 2.2. Mediators between media affinity and cultural identification

Based on the first set of hypotheses, we further examined the mediating effect of Cantonese media on Chinese cultural identification.

For Malaysian Chinese, affinity to Cantonese media affects Chinese cultural identification through the mediation of the perceived value of Cantonese media (Louie, 2004). The “educational and entertaining” content of Cantonese media can effectively enhance the Chinese cultural emotions of the audience. Cantonese two-part sayings, proverbs, and poetry are closely related to humor and culture. Thus, Cantonese media can meet the entertainment needs of Chinese people. The content conveys feelings and ideas, passes on Chinese culture, and stimulates emotions about China and being Chinese. Through exposure to Cantonese media content, Malaysian Chinese have improved their knowledge and understanding of Chinese language, customs, festivals, and food culture. Overseas Chinese learn Cantonese and inherit Chinese language and culture by watching Hong Kong Television Broadcasts Limited (TVB) dramas. Local Cantonese radio stations in Malaysia have long held an industry tradition of imparting inherited Chinese cultural knowledge. For example, Cantonese radio dramas have always been the “signature programs” of the 988 FM radio station. Many Cantonese radio dramas are based on traditional Chinese cultural themes. Talk shows and music programs are the most popular, with Chinese food culture, entertainment, customs and habits, and classical Chinese music also featuring heavily in programs. Cantonese media merges with Chinese culture and pays attention to Chinese traditions, quietly influencing the perception of Chinese culture.

Cantonese media can promote the formation of Chinese cultural cognition, thereby positively impacting on Chinese cultural identification (Sun, 2010). When ethnic minorities are exposed to the mainstream cultural environment of a multi-ethnic country, they may become more inclined to expand their social connections away from the mainstream culture (Kumar, 2018). The more exposed they are to ethnic cultures, the more inclined they may be to expand their connections with ethnic cultural and social groups, thereby promoting ethnic cultural cognition and behavior (Ting-Toomey, 1981). At the same time, people may derive spiritual pleasure from entertainment media and an enhanced cultural identity (González-Avella et al., 2007; Cunningham and Craig, 2019). Chinese Americans’ perception of culture is a type of knowledge acquisition and a channel to connect with contemporary society, enabling them to integrate into an imaginary community based on a common culture (Phillips, 2002). This integration helps relieve the anxiety of daily life (Ostic et al., 2021), build a community of media circles and interests, and develop cultural cognition in the social network connected by Cantonese media (Huang et al., 2021). Social activities further create a cultural environment that allows Malaysian Chinese to develop social connections and enhance their cognition of shared culture (Wan and Chew, 2013).

At the level of psychological path research, a preference for Cantonese media will increase the perceived value of Chinese culture, and then promote its cognition, strengthening the sense of identity with Chinese culture. Ethnic Chinese have transformed the cultural heritage of “imagining China” into a positive, spiritual connection with their homeland. As Edward Said noted that imagined geography and history help dramatize the difference between nearby and distant places and intensify the sense of self (Said, 1977). Malaysian Chinese enjoy Cantonese media and immerse themselves in it. The cultural imagination and memory aroused in this process narrow the distance between Malaysian Chinese and traditional Chinese culture and build a cultural and spiritual home for Malaysian Chinese.

Therefore, we proposed a second set of hypotheses:

*H4*: The emotional affinity to Cantonese media affects Chinese cultural identification through the perceived value of Cantonese media, and the perceived value of Cantonese media plays a mediating role.

*H5*: The emotional affinity to Cantonese media affects Chinese cultural identification through Chinese cultural cognition, and Chinese cultural cognition plays a mediating role.

*H6*: The emotional affinity to Cantonese media affects Chinese cultural identification through the chain mediation of the perceived value of Cantonese media and Chinese cultural cognition.

### 2.3. Moderators of cultural identity

Cultural knowledge disseminated in public spaces strongly influences individuals’ understanding of traditional or heritage culture. This serves to strengthen their cultural identity of origin, especially for older people (Wan and Chew, 2013). There is limited systematic research on the influence of gender and other demographic variables on Chinese cultural identification. Therefore, we added three moderating variables – gender, age, and the generation of Chinese – to test whether there are structural differences in the impact of Cantonese media on Chinese cultural identification by groups with different characteristics.

*H7*: Gender has a moderating effect on the path of Cantonese media emotional affinity on Chinese cultural identity.

*H8*: Age has a moderating effect on the path of Cantonese media emotional affinity on Chinese cultural identity.

*H9*: The generation of Chinese has a moderating effect on the path of Cantonese media emotional affinity on Chinese cultural identity.

**Figure 1:**
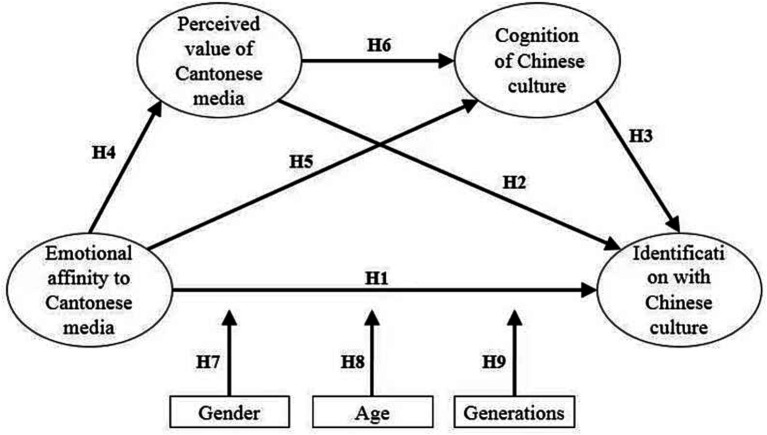
Research framework.

The literature review provided a theoretical foundation for this study’s in-depth exploration of the construction of cultural identity, the level of individual psychology, and the level of collective culture. Although studies have consistently pointed out that Cantonese media provides multidimensional value in the process of constructing Chinese cultural identity, how Cantonese media affects cultural identity is still at the early stages of qualitative analysis and theoretical discussion. The present study proposed the above research hypotheses and a path map of the influence of Cantonese media emotional preferences on Chinese cultural identity (see Figure 1).

## 3. Materials and methods

### 3.1. Sampling

A survey covering Cantonese media use, Chinese cultural identification, and demographic data was administered in September 2021 by research team members from the Department of Southeast Asian Studies, University of Malaya, Malaysia, and the Department of Journalism, Xiamen University, Malaysia Branch. Before distributing the survey on a large scale, we invited three experts – a Malaysian historian, a linguist, and a communication scientist – to review and modify it as necessary. We also conducted field surveys and identified 10 respondents to participate in in-depth interviews exploring vivid humanistic themes behind the data.

The respondents filled in the questionnaire based on their interpretation of the questions. Some participants had only received an English-language education and could not read Chinese. Thus, the questionnaire was available in both languages, and respondents received a monetary incentive to complete it. The respondents’ details were confirmed to ensure they met the research requirements.

A multistage sampling strategy was used to select a representative sample of residents aged 15 years or older from five districts of Kuala Lumpur: Bukit Bintang, Setiawangsa, Kepong, Lembah Pantai, and Cheras. We used equal distance sampling to choose 2000 households, from which ethnic Chinese households were selected. A Kish grid was then used to randomly select participants over 15 years of age from these households, providing 479 valid samples. The questionnaires were distributed, completed, and collected on the spot. Meanwhile, 163 ethnic Chinese participants aged 14 or younger were randomly selected from five types of Chinese-medium institutions in Kuala Lumpur: Chinese primary schools, national and international primary schools, and independent and national secondary schools. These respondents were selected based on their school ID numbers. They received hard copies of the questionnaires and were helped by teachers to complete the surveys. Ultimately, we received a total of 642 valid questionnaires. The descriptive information of the group characteristics of respondents is presented in Table 1. The ethnic Chinese participants in our study were mainly female, accounting for 51.5% of the sample. The percentage of adults was 60.1%, and the proportions of new and old immigrants were very similar (old immigrants: 48.9%). The number of respondents who received only English-language education was very low, accounting for 5.5% of the total population.

**Table 1:** The descriptive information of respondents (*N* = 642).

Variables	Items	Frequency	%
Gender	Male	311	48.5
	Female	331	51.5
Age	Minor	256	39.9
	Adult	386	60.1
Generation of ethnic Chinese	New immigrants	328	51.1
	Old immigrants	314	48.9
Received language education	Both Chinese and English	607	94.5
	Only English	35	5.5

### 3.2. Measurements

Table 2 shows the content of the structured questionnaire, along with the sources, and details of each measure.

**Table 2:** The sources and details of measurements.

Factors	Items	Range	Sources
Identification with Chinese culture	7 items How do you feel about Chinese culture? I really agree with Chinese culture I appreciate Chinese culture I am proud of Chinese culture I usually do things according to Chinese culture It is important for me to maintain and develop Chinese culture I enjoy living in an environment with Chinese culture I am willing to promote Chinese culture	1–5, the higher scores, the more identification with Chinese culture.	Adapted from Bhugra et al. (1999) and Wu and Shi (2014)
Emotional affinity to Cantonese media	7 items How much do you like the following Cantonese media? Cantonese pop music Cantonese radio Cantonese TV programs Cantonese film Cantonese TV series Cantonese opera Online Cantonese short video	1–5, the higher scores, the stronger emotional affinity to Cantonese media	Adapted from Kim et al. (2013)
Perceived value of Cantonese media	16 items divided into 4 dimensions by item parceling method. How do you feel about Cantonese media? 1. Entertainment value: Let me relieve stress Make me laugh Give me positive energy Make me relaxed 2. Culture value: Let me know about Cantonese culture Let me know about Guangdong culture Let me know about Hong Kong culture Let me know about Malaysian Chinese culture 3. Instrumental value: Let me learn Cantonese Let me keep my Cantonese skills Needed for work or study abroad 4. Social value Let me know about the latest news Let me know about hot topics Let me get sense of companionship Let me participate in Chinese community activities Let me make friends.	1–5, the higher scores, the stronger perceived value	Adapted and integrated from Abbas Naqvi et al. (2020), Albert (2021), Swaid and Wigand (2012), and Wan and Chew (2013)
Cognition of Chinese culture	9 items divided into 2 dimensions by item parceling method. How much do you know about the following festivals, characters, and ideas? 1.Traditional Chinese festivals: Lunar New Year Lantern Festival Tomb-Sweeping Day Dragon Boat Festival The Ghost Festival The Mid-Autumn Festival 2. Traditional Chinese thoughts Culture of filial duty Confucius, Li Bai, Sun Yat-sen and others Confucianism	1–5, the higher scores, the more cognition of Chinese culture	Adapted from Hao (2019)
Gender, age and generation of ethnic Chinese	3 items	0–1, male or female; minors or adults; new or old ethnic Chinese	Self-made

#### 3.2.1. Identification with Chinese culture (dependent variable)

This refers to overall affirmation and recognition of the values, standards, morality, customs, and other aspects embodied by Chinese culture (Zhang, 2005). We adapted Bhugra et al.’s (1999) measure of identification with Asian culture, and Wu and Shi’s (2014) measure of identification with Chinese culture. Seven items were selected with a 5-point Likert scale (1 = very inconsistent, 5 = very consistent) to measure and establish the degree of identification with Chinese culture. The items evaluated the extent to which participants identify with Chinese culture, appreciate Chinese culture, are proud of Chinese culture, act according to Chinese culture, attach importance to maintaining and developing Chinese culture, enjoy a Chinese cultural environment, and are willing to promote Chinese culture. The standardized factor loading of the questions was 0.823–0.878 (see Table 3). For internal consistency reliability, Cronbach’s α = 0.939. The composite reliability was CR = 0.950 and the convergent validity AVE = 0.732. The factor of identification with Chinese culture has good reliability and convergent validity (Fornell and Larcker, 1981).

**Table 3:** Factor characteristics of the measurement model: reliability and convergence validity.

Factors	Items	Std. Factor Loading	*p*	Cronbach’s α	CR	AVE
Emotional affinity to Cantonese media	E1	0.805	<0.001	0.903	0.923	0.633
	E2	0.773	<0.001			
	E3	0.830	<0.001			
	E4	0.731	<0.001			
	E5	0.815	<0.001			
	E6	0.784	<0.001			
	E7	0.826	<0.001			
Perceived value of Cantonese media	Entertainment	0.832	<0.001	0.838	0.892	0.673
	Culture	0.832	<0.001			
	Instrumental	0.766	<0.001			
	Social	0.850	<0.001			
Cognition of Chinese culture	Holidays	0.903	<0.001	0.649	0.848	0.736
	Ideology	0.810	<0.001			
Identification with Chinese culture	C1	0.876	<0.001	0.939	0.950	0.732
	C2	0.874	<0.001			
	C3	0.878	<0.001			
	C4	0.829	<0.001			
	C5	0.856	<0.001			
	C6	0.823	<0.001			
	C7	0.851	<0.001			

Std. factor loading, Standardized factor loading; CR, Composite reliability; AVE, Average variance extracted.

#### 3.2.2. Emotional affinity to Cantonese media (independent variable)

This variable measured the degree of fondness individuals have toward Cantonese media. We focused on the main Cantonese media used by Malaysian Chinese in their daily lives, and referred to Kim et al.’s (2013) measure of fondness for social media. Seven items were presented using a Likert scale with five points where 1 = dislike and 5 = like very much. The items included Cantonese pop music, Cantonese movies, Cantonese TV plays, Cantonese radio and TV programs (Guangdong, Hong Kong, and Malaysia), and online Cantonese audio/video. The seven items jointly established the degree of emotional affinity to Cantonese media. The standardized factor loading was 0.731–0.830, Cronbach’s α = 0.903, CR = 0.923, and AVE = 0.633. The degree of emotional affinity to Cantonese media has good reliability and convergent validity.

#### 3.2.3. Perceived value of Cantonese media (mediating variable)

In general, perceived media value can be divided into four dimensions: entertainment value (Abbas Naqvi et al., 2020), cultural value (Albert, 2021), tool value (Swaid and Wigand, 2012), and social value (Wan and Chew, 2013). Based on our literature review, the study first measured the four dimensions of media perceived value, using 16 items rated on a five-point Likert scale (1 = very inconsistent, 5 = very consistent). Of these 16 items, four focused on entertainment value (e.g., relieving stress and relaxing); four evaluated cultural values (e.g., learning about Guangdong and Malaysian cultures); three were on tool value (e.g., maintaining Cantonese ability and work needs), and five items centered on social value (e.g., having company and making friends). The internal consistency reliability of each dimension was 0.910, 0.915, 0.728, and 0.897, respectively. Second, to ensure the model parameter estimation was stable, we adopted the item parceling method to make a weighted total average of the score of the items within each dimension (Hall et al., 1999; Wu and Wen, 2011). This average score index represents the score of each dimension. Finally, after processing by item parceling, four indexes were used to establish the factor of perceived value of Cantonese media. The index normalization factor load was 0.766–0.850, Cronbach’s α = 0.838, CR = 0.892, and AVE = 0.673. The factor of perceived value of Cantonese media has good reliability and convergent validity.

#### 3.2.4. Cognition of Chinese culture (mediating variable)

Cultural cognition refers to a mental activity process of gaining knowledge through an individual’s information processing of the contents, values, and viewpoints of a specific culture (Hong et al., 2000), and indicates the degree of understanding of this culture. Chinese culture includes folk customs, thought, costumes, literature, art, and many other aspects. We referred to Hao’s (2019) measure of cognition of Chinese culture among Southeast Asian ethnic Chinese, and developed nine items with a five-point Likert scale (1 = do not know at all, 5 = know very well) to establish participants’ cognition of Chinese culture. The factor can be divided into two dimensions, each including six questions about traditional festivals (e.g., Lunar New Year, Lantern Festival, and Dragon Boat Festival) and three questions about traditional Chinese thoughts (e.g., culture of filial duty, historical figures, and Confucianism). The internal consistency reliability was 0.918 and 0.841, respectively. The item parceling method was again adopted to make a weighted total average of the score of the items within each dimension, and finally, two dimension indexes were used to establish the factor of cognition of Chinese culture. The index standardized factor loading were 0.903 and 0.810, respectively, Cronbach’s α = 0.649, CR = 0.848, and AVE = 0.736. The factor of cognition of Chinese culture has good reliability and convergent validity.

#### 3.2.5. Gender, age, and generation of ethnic Chinese (moderating variables)

Participant gender was either male =0 or female =1. For age, we established 18 years as a boundary: participants younger than 18 were regarded as minors (=0), and participants aged 18 years or older were considered adults (=1). Zhu and Dai (2002) pointed out that it will take 2–3 generations for overseas ethnic Chinese to fully integrate into the local society and complete the transition from “transient” to “settling.” Therefore, this paper classified the 1st – 3rd generations of Malaysian Chinese as “new ethnic Chinese” (= 0), and the 4th generation and above as “old ethnic Chinese” (=1).

We aimed to investigate the discriminant validity of the four factors, namely, emotional affinity to Cantonese media, perceived value of Cantonese media, cognition of Chinese culture, and identification with Chinese culture. Table 4 shows that using Fornell and Larcker’s criterion (Cohen, 1988), the AVE square root of each factor exceeds its correlations with other factors. At the same time, the heterotrait-monotrait (HTMT) ratio of correlations between every two factors is below 0.85. Therefore, there is good discriminant validity between factors (Urbach and Ahlemann, 2010). In summary, all factors had good reliability and validity in this research.

**Table 4:** Factor characteristics of the measurement model: discriminative validity.

Factors	Discriminative validity			
	EACM	PVCM	CCC	ICC
EACM	***0.796***			
PVCM	0.691 (0.789)	***0.820***		
CCC	0.409 (0.526)	0.441 (0.579)	***0.858***	
ICC	0.497 (0.532)	0.554 (0.616)	0.529 (0.662)	***0.855***

EACM, emotional affinity to Cantonese media; PVCM, perceived value of Cantonese media; CCC, cognition of Chinese culture; ICC, identification with Chinese culture. The figures (bold and italic font) on the diagonal are the AVE square root values; the figures in the inferior triangle are the Pearson correlation coefficients; and the values in the “()” are the HTMT values.

Data came from interviewees’ responses to the same questionnaire. Common method variance (CMV) may occur in such circumstances leading to a deviation in the real relationship between variables (Podsakoff et al., 2003). Thus, measures to investigate and mitigate CMV were put into place. First, when designing the questionnaire, we adopted the item meaning concealment method (DeVellis, 2016) to prevent CMV. Second, before the model analysis, an ex-post detection was performed for CMV with the following measures. (1) We adopted Harman’s one-factor method of putting all variables in the factor analysis and abstracting one common factor (not transferred). The analysis showed that the variance explained by the one common factor was 46.21%. Taking 50% as the criterion (Ylitalo, 2009), we made a preliminary judgment that CMV does not exist. (2) We applied the unmeasured latent method construct (Wu et al., 2012) with SmartPLS software. The average substantial explained variation (*R1^2^*) and the CMV (*R2^2^*) were calculated as 0.697 and 0.013, respectively. Therefore, *R1^2^*:*R2^2^* = 55:1. Thus, CMV is not a serious concern in this research (Liang et al., 2007).

### 3.3. Statistical analyses

We used SmartPLS 3.3.2 (SmartPLS GmbH, Boenningstedt) for statistical analysis. Based on the variance of the observed variables in the structural equation model, we employed partial least squares (PLS), principal component analysis, and multiple regression analysis to estimate the model parameters and maximize the model’s predictive power. PLS differs from structural equation model analysis based on covariance (Chin and Newsted, 1999) and has some unique advantages: (1) Data are not required to conform to multivariate normal distribution; (2) Multidimensional complicated structural equation models can be handled, and (3) It is suitable for model prediction, maximizing the interpreted power of endogenous variables (Henseler et al., 2009). PLS statistical analysis methods and application software have become widely used in scientific research (Ringle et al., 2012). This study adopted the SmartPLS software for the following reasons: (1) The structural equation model established in the study is complicated, involving mediation and moderation effects; (2) The study focused on the influence of factors such as emotional affinity to Cantonese media on identification with Chinese culture among Malaysian Chinese. Using PLS used in conjunction with the proposed theoretical framework enabled us to maximize the power to interpret Chinese cultural identification.

## 4. Results

### 4.1. Descriptive statistics

The indications of each factor of the structural equation model were summed and averaged (see Table 5). The emotional affinity of ethnic Chinese to Cantonese media was above average. The mean value was 3.281 (highest: 5) and the standard deviation was 0.867. Similarly, the ethnic Chinese held a generally positive attitude toward the perceived value of Cantonese media (*M* = 3.512, SD = 0.711), had a general understanding of Chinese culture (*M* = 3.640, SD = 0.711), maintained a relatively strong recognition of Chinese culture (*M* = 4.051, SD = 0.707), and identified in an overall positive way with Chinese culture.

**Table 5:** Descriptive statistics of factors.

Factors	*M*	SD	Min	Max
EACM	3.281	0.867	1.000	5.000
PVCM	3.512	0.711	1.000	5.000
CCC	3.640	0.711	1.195	5.000
ICC	4.051	0.707	1.000	5.000

EACM, emotional affinity to Cantonese media; PVCM, perceived value of Cantonese media; CCC, cognition of Chinese culture; ICC, identification with Chinese culture; M, mean; SD, standard deviation.

### 4.2. Structural path and mediating effects

The path coefficients between factors were estimated and inspected first, based on the research framework. The PLS software can only estimate standardized path coefficients. Thus, we adopted the bootstrapping method for 5,000 repeated samples to obtain the standard error and calculate the *p-*values. The bias-corrected method was used to obtain the upper and lower limits of the path coefficient in the 95% confidence interval. Table 6 shows that the emotional affinity to Cantonese media, perceived value of Cantonese media, and cognition of Chinese culture all have a positive influence on the identification with Chinese culture–with standardized path coefficients *β* of 0.153 (*p* < 0.001), 0.302 (*p* < 0.001) and 0.333 (*p* < 0.001), respectively. The more strongly participants liked Cantonese media, the more positively they rated its perceived value. When Chinese culture was well-understood, participants identified more strongly with it. Emotional affinity to Cantonese media has a positive influence on the perceived value of Cantonese media and the cognition of Chinese culture, with path coefficients of *β =* 0.691 (*p* < 0.001) and 0.205 (*p* < 0.001), respectively. The greater participants’ liking for Cantonese media, the more useful they consider it for themselves and the more deeply they understand Chinese culture. At the same time, the perceived value of Cantonese media also had a positive influence on the cognition of Chinese culture. The more valuable Cantonese media was considered to be, the more deeply Chinese culture was understood (*β* = 0.298, *p* < 0.001). For the six path coefficients, the estimated value range with a 95% confidence interval did not contain 0. Thus, there is strong evidence to show the existence of relationships between factors. In the structural equation model, the identification with Chinese culture is a dependent variable (*R*^2^ = 0.419), showing that the model can explain 41.9% of the variance of the identification with Chinese culture. Therefore, the model has moderate explanatory power, and its overall validity is acceptable (Hair et al., 2021). The model prediction correlation was *Q*^2^ = 0.300 (greater than 0), indicating the model has good predictive power (Tenenhaus et al., 2005).

**Table 6:** Structural model path coefficients, indirect effects, and total effect.

Path coefficients	Estimate	SD	T	*p*	Bootstrapping	
					Bias-Corrected	
					2.5%	97.5%
EACM -> ICC	0.153	0.043	3.531	<0.001	0.067	0.236
PVCM -> ICC	0.302	0.046	6.626	<0.001	0.213	0.392
CCC -> ICC	0.333	0.037	9.009	<0.001	0.260	0.404
EACM -> PVCM	0.691	0.024	28.863	<0.001	0.638	0.732
EACM -> CCC	0.205	0.049	4.193	<0.001	0.107	0.296
PVCM -> CCC	0.298	0.048	6.174	<0.001	0.204	0.391
Indirect effects						
PVCM (Mediator)						
EACM -> ICC	0.208	0.033	6.276	<0.001	0.146	0.275
CCC (Mediator)						
EACM -> ICC	0.068	0.019	3.585	<0.001	0.046	0.098
Chain mediation: PVCM (Mediator1) -> CCC (Mediator2)						
EACM -> ICC	0.069	0.014	5.046	<0.001	0.074	0.136
Total effect						
EACM -> ICC	0.498	0.034	14.599	<0.001	0.428	0.562

EACM, emotional affinity to Cantonese media; PVCM, perceived value of Cantonese media; CCC, cognition of Chinese culture; ICC, identification with Chinese culture; SD, Standard Deviation.

The estimated value of the total effect points of the influence of emotional affinity to Cantonese media on identification with Chinese culture was *TE* = 0.498 (*p* < 0.001). The direct effect was *DE* = 0.153 (*p* < 0.001). Taking perceived value of Cantonese media as a mediator variable, in the path of emotional affinity to Cantonese media influencing identification with Chinese culture, the perceived value of Cantonese media has a significant mediating effect (see Table 6). The estimated value of indirect effect points was *IE* = 0.208 (*p* < 0.001) and the estimated value range with a 95% confidence interval (0.146–0.275) did not contain 0, showing that the influence of emotional affinity to Cantonese media on identification with Chinese culture is partly realized through the mediating effect of the perceived value of Cantonese media. Similarly, cognition of Chinese culture had a significant mediating effect on relations between emotional affinity to Cantonese media and identification with Chinese culture, *IE* = 0.068 (*p* < 0.001). The estimated value range with a 95% confidence interval (0.046–0.098) did not contain 0. Further exploration shows that in the influence of emotional affinity to Cantonese media on identification with Chinese culture, the chain mediation pathway *via* perceived value of Cantonese media and cognition of Chinese culture also has a significant effect, *IE* = 0.069 (*p* < 0.001). The estimated value range with a 95% confidence interval (0.074–0.136) did not contain 0. Thus, participants’ fondness of Cantonese media can further influence the cognition of Chinese culture through perceiving the value of Cantonese media and, eventually, positively influencing identification with Chinese culture.

### 4.3. Moderating effect

We undertook group comparisons of gender (male, female), age (minor, adult), and generations of ethnic Chinese (new, old) as moderator variables for investigating the moderating effect of each variable on the model. Table 7 shows that gender had no moderating effect on any paths in the model. Age had some moderating effects. Differences between adults and minors were noted in the influence of emotional affinity to Cantonese media on perceived value of Cantonese media and cognition of Chinese culture, and in the mediating effect of the perceived value of Cantonese media and cognition of Chinese culture. Adults’ perceived value of Cantonese media tends to be more influenced by Cantonese media. The difference of the path coefficients (adult–minor) was *β_diff_ = 0*.159, *p* = 0.002. In contrast, the minors’ degree of fondness for Cantonese media more strongly affects cognition of Chinese culture than adults’ degree of fondness (*β_diff_ =* −0.287, *p* = 0.004), and this moderating effect also has a significant influence on the mediating effect of cognition of Chinese culture (*IE_diff_* = −0.097, *p* = 0.008). The mediating effect of cognition of Chinese culture is stronger in minors than adults. Finally, the total effect of emotional affinity to Cantonese media on identification with Chinese culture was stronger in adults than in minors. The difference of the total effect values was *TE_diff_* = 0.126, *p* = 0.005. Given the same emotional affinity to Cantonese media, adults’ degree of identification with Chinese culture is higher than that of minors. There were no differences in the model regarding new and old ethnic Chinese, and generation had no moderating effect.

**Table 7:** Comparison of path coefficients and mediating effects of subgroups.

Path coefficients	Female – Male		Adult – Minor		Old – New Generation	
	Difference	*p*	Difference	*p*	Difference	*p*
EACM -> ICC	−0.065	0.231	0.117	0.182	−0.043	0.638
PVCM -> ICC	0.009	0.921	−0.044	0.634	0.129	0.167
CCC -> ICC	−0.056	0.454	0.003	0.967	0.001	0.998
EACM -> PVCM	0.044	0.329	0.159	**0.002**	−0.003	0.959
EACM -> CCC	0.171	0.079	−0.287	**0.004**	0.185	0.064
PVCM -> CCC	−0.110	0.265	0.327	**<0.001**	−0.050	0.595
Indirect effects						
PVCM (Mediator)						
EACM -> ICC	0.020	0.763	0.015	0.802	0.088	0.193
CCC (Mediator)						
EACM -> ICC	−0.054	0.172	−0.097	**0.008**	0.061	0.089
Chain mediation: PVCM (Mediator1) -> CCC (Mediator2)						
EACM -> ICC	−0.033	0.245	0.092	**0.001**	−0.012	0.669
Total effect						
EACM -> ICC	−0.033	0.631	0.126	**0.005**	0.094	0.178

EACM, emotional affinity to Cantonese media; PVCM, perceived value of Cantonese media; CCC, cognition of Chinese culture; ICC, identification with Chinese culture; the bold figures mean *p* < 0.05.

## 5. Discussion

Malaysian Chinese evaluated and perceived Cantonese media in a positive way, and their emotional affinity to Cantonese media and its perceived value were at a moderate to high level. Malaysian Chinese have some knowledge and understanding of Chinese culture and retain a strong sense of identification with it.

The stronger participants’ preference for Cantonese media (H1 supported), the more they find Cantonese media valuable (H2 supported). The more they understand Chinese culture (H3 supported), the stronger their identification with Chinese culture. Emotional affinity to Cantonese media affected the perceived value of Cantonese media, which, in turn, indirectly affected Chinese cultural identity, with perceived value of Cantonese media as a mediator (H4 supported). Similarly, emotional affinity to Cantonese media can indirectly influence Chinese cultural identification through Chinese cultural cognition, which plays a partial mediating role (H5 supported). Cantonese media affective preferences can also indirectly influence Chinese cultural identification through the chain mediation of perceived value of Cantonese media and Chinese cultural cognition (H6 supported).

Gender had no moderating effect on the pathways between emotional affinity to Cantonese media and Chinese cultural identification (H7 rejected). Age had some effects (H8 supported): emotional affinity to Cantonese media had a stronger positive impact on perceived value of Cantonese media in adults, but its impact on Chinese cultural cognition was stronger in minors. Through multiple mediations, adults’ emotional affinity to Cantonese media can ultimately influence their Chinese cultural identification more strongly. Adult preferences for Cantonese media can be transformed into identification with Chinese culture more effectively. Generation had no effects (H9 rejected): the pathways and effect of the medium emotional affinity on Chinese cultural identification remained consistent irrespective of generation.

### 5.1. From Cantonese media affinity to Chinese cultural identification

The total effect of emotional affinity to Cantonese media on Chinese cultural identification was 0.498 (see Table 6, *TE* = 0.498), which has good explanatory power in the structural equation model. Essentially, when individuals enjoy Cantonese media, their Chinese cultural identification is enhanced. Although Cantonese is only a local language in China, the Cantonese-based media has an extremely important influence in shaping Chinese cultural identification among overseas Chinese.

Cantonese media uses cultural memory as a bridge to construct the Chinese cultural identity of Malaysian Chinese. In overseas Chinese communities, Cantonese has an important cultural inheritance function – prominently reflected in cultural carriers such as pop music, film, television, and other entertainment products. Communication scholars have paid attention to the diffusion effects of Cantonese pop music, Hong Kong film and television dramas, and Cantonese opera in Malaysia, affirming the entertainment and cultural value of these media in the 21st century. Cantonese film and television dramas have brought pleasure to the relatively undernourished spiritual life of Malaysian Chinese, and Cantonese pop music is a tool for Malaysian Chinese to alleviate their nostalgia. Cantonese media, in a range of popular audio and visual forms, enhances Malaysian Chinese’ love for such media and ongoing identification with Chinese culture (Kang et al., 2021).

Love of mother tongue media can enhance immigrants’ perception of their native culture and foster their Chinese cultural identity (Seto and Martin, 2019). For example. Hong Kong Cantonese martial arts films are a source of entertainment for Malaysian Chinese, connecting the imaginary space between “real life” and nostalgic emotions. Hong Kong films influence the cultural identity of overseas Chinese because their discrete narratives present globally dispersed cultural memories on the screen, further shaping the global Chinese community. In combination, a shared love of Cantonese media, familiar movies, and Cantonese songs have created a cultural community among Malaysian Chinese.

### 5.2. The mediating roles of perceived value of Cantonese media and cognition of Chinese culture

Converting the emotional affinity to Cantonese media into identification with Chinese culture is not a simple, linear path from A to B, but a complex path mechanism. Emotional affinity to Cantonese media can influence Chinese cultural identification through the perceived value of Cantonese media and Chinese cultural cognition as mediators. Moreover, media value and cultural cognition sequentially mediated between emotional affinity and cultural identity. In this study, the total indirect effect of perceived value of Cantonese media and Chinese cultural cognition was 0.345 (see Table 6, *IE* = 0.208 + 0.068 + 0.069).

Cantonese media enhances Chinese cultural identity through the mediating influence of perceived value and cultural cognition. Self-determination theory proposes that media users have autonomous needs, including the need for spiritual satisfaction and psychological relaxation (Cheng and Leung, 2020). A preference for media makes individuals more likely to use media to relieve stress and relax, and to perceive Chinese culture in that process(Jamil et al., 2022). Cantonese media, especially songs and TV dramas, can be very relaxing. Enjoying their favorite media, Malaysian Chinese can subtly infiltrate Chinese cultural cognition and effectively build a Chinese cultural identity.

Cantonese culture is an important factor in predicting Chinese cultural identity. Cantonese culture is a regional symbol of Chinese culture and of great significance to the construction of Chinese community consciousness. In particular, original hometown culture helps preserve traditional cultural characteristics and cultural festivals of the ancestors, thereby strengthening geographical identity. Chinese community activities organized through online media focus on protecting the use of the Cantonese language, ensuring that Chinese history and culture are shared and friends made with those who have been through similar diaspora experiences. In the process, media preference promotes Chinese cultural cognition by enhancing perceived value, further shaping group cultural identity, building a network of interest and affinity, and strengthening cultural identity through ethnic ties.

### 5.3. Insights from age groups on the promotion of Cantonese media

Comparing between age groups, adults’ (vs. minors’) emotional affinity to Cantonese media had a stronger impact on their perceived value of Cantonese media (*β_diff_* = 0.159), whereas minors’ (vs. adults’) emotional affinity to Cantonese media had a stronger effect on their cognition of Chinese culture (*β_diff_* = −0.287). The idiosyncratic function of Cantonese media for adult audiences is reflected in the practical meaning of the media to everyday life, while Cantonese media for minors provides knowledge of Chinese culture. In general, emotional affinity to Cantonese media is more likely to enhance Chinese cultural identification in adults than in minors (see Table 7, *TE_diff_* = 0.126).

In terms of media influence on the audience, young people are in the stage of exploring knowledge and are sensitive to understanding Chinese culture conveyed by Cantonese media. The more they enjoy Cantonese media, the more they will understand Chinese culture. At the same time, the development of Chinese education in Malaysia has its own complete system characterized by Chinese language education from childhood to university. More than 90% of Chinese primary school students are enrolled in Chinese primary schools. The influence path of cognition played an obvious moderating role in the study. In comparison, adults have to earn a living and live in many places around the world. They value the instrumental and entertainment value of media to improve themselves and enrich their daily lives. Thus, their emotional preferences for Cantonese media have a significantly stronger effect on the perceived value of Cantonese media. From the perspective of media effectiveness, Cantonese media distinguishes differences in media content needs of Chinese of different age groups to formulate more refined communication content. In the practice of international communication in Cantonese for adults, information application, innovative expression, entertainment, and all experiences should be valued. For minors, the dissemination and shaping of traditional cultural knowledge should be emphasized.

Young Malaysian Chinese are in an important period of cultural identity construction but follow a different approach from the active and self-conscious construction methods of adult Chinese Americans. The cultural identity construction of young Malaysian Chinese is often diluted by family and social life – an inherited cultural identity construction (Christensen, 2019; Lin et al., 2019). From this perspective, it is clear why adults are more likely than minors to enhance their Chinese cultural identity through Cantonese media.

## 6. Conclusion and limitations

Media is the carrier of culture, and the love of Cantonese media promotes the Chinese cultural identity of ethnic Chinese. Through the empirical data of Malaysian Chinese, this study revealed the psychological path of the influence of native-language media on cultural identity: through fondness for Cantonese media, the value of native language is perceived, and cultural memory is awakened to subtly promote cultural identity. The international communication of Cantonese media cultivates an emotional preference of Chinese groups for Cantonese media. These groups pay attention to the effective improvement of the entertainment value, cultural value, and instrumental value of Cantonese media. Cantonese is used as a medium for international communication, and the media is instrumental in building a global Chinese cultural community. Cantonese media should be empowered for greater cultural dialog and knowledge dissemination.

Our study responds to classic research on immigration in the United States. Immigrants communicated and interacted with society through ethnic media, which helped them adapt to local society and integrate more quickly into local life. The “stranger” is not a wanderer who comes today and leaves tomorrow, but a wanderer who comes today and stays tomorrow. Phenomenologist Alfred Schutz pointed out that “strangers” seek balance between local and native cultures. In *The Immigrant Press and Its Control*, Robert Ezra Park (1922) suggested that immigrants come into contact with and understand mainstream society through the ethnic language. Stonequist (1935) similarly noted that immigrant newspapers help to preserve the ethnic culture; through the language of the ethnic group, new immigrants can adapt to urban life, understand the local mainstream society, alleviate homesickness, and thus integrate smoothly into American society. Human mobility is undoubtedly becoming more convenient and cultural exchange more accessible, whether in the Chinese cultural circle in Southeast Asia, San Francisco’s Chinatown, Guangdong’s African community, or London’s Koreatown. Against this background, the mother-language media and cultural identity of diasporas will be increasingly explored by academics.

The study has several limitations. First, our sample did not include all states in Malaysia but only Kuala Lumpur, the most concentrated area of Chinese. Second, compared with the United States, Europe, Japan, and other countries, most Malaysian Chinese are older Fujian and Cantonese immigrants who have “gone down to Nanyang” from the southeast coast of China. These groups have distinct dialect characteristics and geo-cultural characteristics. The sample was limited to Malaysian Chinese, and follow-up work will involve more ambitious cross-country comparative studies. Third, as more residents of mainland China’s inland provinces have chosen to emigrate in recent years, Mandarin-language media has also gradually emerged overseas. Whether the Mandarin media’s identification with Chinese culture has the same influence and mechanism as that of Cantonese media is worthy of subsequent multilingual media comparison. In short, the application of structural equation modeling in this study has broadened and enhanced the research path and application value of quantitative research in communication psychology. The study provides a useful reference and guidance for developing subsequent related research. The intersection of psychology and communication can enrich the knowledge base of quantitative communication and promote the development of interdisciplinary research.

## Data availability statement

The raw data supporting the conclusions of this article will be made available by the authors, without undue reservation.

## Ethics statement

The studies involving humans were approved by Ethics Committee of the School of Public Administration of Guangzhou University (No.HBR20210121). The studies were conducted in accordance with the local legislation and institutional requirements. Written informed consent for participation in this study was provided by the participants’ legal guardians/next of kin.

## Author contributions

NL, YP, and YX contributed to conception, design of the study, and wrote the first draft of the manuscript. YP and YX organized the database. NL and YX performed statistical analysis. NL, TC, YP, and YX wrote sections of the manuscript. All authors contributed to manuscript revision, and have read and approved the submitted version.

## Funding

This work was supported by the National Social Science Fund of China (Project No. 21CXW030) and the National Office for Philosophy and Social Sciences.

## Conflict of interest

The authors declare that the research was conducted in the absence of any commercial or financial relationships that could be construed as a potential conflict of interest.

## Publisher’s note

All claims expressed in this article are solely those of the authors and do not necessarily represent those of their affiliated organizations, or those of the publisher, the editors and the reviewers. Any product that may be evaluated in this article, or claim that may be made by its manufacturer, is not guaranteed or endorsed by the publisher.
